# Role of complement in IgA nephropathy

**DOI:** 10.1007/s40620-015-0245-6

**Published:** 2015-11-13

**Authors:** Mohamed R. Daha, Cees van Kooten

**Affiliations:** Department of Nephrology, Leiden University Medical Center, Albinusdreef 2, 2333ZA Leiden, The Netherlands

**Keywords:** IgA nephropathy, Complement, MBL, C4d

## Abstract

Immunoglobulin A nephropathy (IgAN) is characterized by the deposition of IgA in the mesangium of glomeruli. This mesangial IgA has been found to consist mainly of polymeric IgA1 which drives the activation of the mesangial cells and results in excessive production of several inflammatory mediators. The activation of mesangial cells is amplified by the ability of IgA to activate the complement system, originally thought to occur mainly via the alternative pathway of complement. However more recent studies indicate that lectin pathway involvement has a strong association with progression of renal disease. In this review we summarize the contribution of complement to the IgA- mediated inflammatory process.

## Introduction

Immunoglobulin A nephropathy (IgAN) is characterized by the deposition of IgA in the mesangium of glomeruli. This mesangial IgA has been found to consist mainly of polymeric IgA1 which drives the activation of the mesangial cells and results in excessive production of several inflammatory mediators. The activation of mesangial cells is amplified by the ability of IgA to activate the complement system, originally thought to occur mainly via the alternative pathway of complement. However, more recent studies indicate that lectin pathway involvement has a strong association with progression of renal disease. In this review, we summarize the contribution of complement to the IgA- mediated inflammatory process.

## The complement system

Complement activation proceeds via three known pathways [1, 2]. Initiation of these pathways occurs by pattern recognition. Activation of the classical pathway takes place after binding of the first component of complement C1 to, for instance, immune complexes. Activated C1 then leads to activation of its natural substrates, C4 and C2, and the generation of activator-bound C4b2a, the classical pathway C3 convertase, which is able to cleave C3 into C3b and C3a. C3b has the ability to attach itself in a covalent fashion to the activator or to neighbouring tissue or cells. The generation of C3b is one of the most important steps in complement activation and function because it allows recognition of foreign pathogens or immune complexes with cellular elements of our defence systems via specific interaction with cellular C3 receptors. There are a number of receptors for activated C3, such as the receptor for C3b (CD35), iC3b (CR3) and for C3dg (CD21). CD35 is mainly found on primate erythrocytes where it plays an essential role in the binding and proper handling of circulating immune complexes. The conversion of activator-bound C3b to iC3b by inhibitors like factor I and H allows a pathogen or immune complex that has been opsonized with iC3b to be recognized by specific phagocytic receptors on, for instance, macrophages or polymorphonuclear leukocytes. This is a very important step in complement activation because it contributes to the elimination of foreign pathogens or self-debris by the innate immune system. Therefore, deficiencies in C3 are associated with different bacterial infections. The further breakdown of activator-bound C3 to C3dg adds another dimension to complement-mediated defence. Antigens that are opsonized with C3dg are recognized to a much better extent by follicular B cells and antigen-presenting cells, leading to initiation of an efficient acquired immune response. It was noted already back in the ‘70 s that C3 activation is essential for an optimal antibody response against foreign antigens. Additionally, several studies during the past decades have shown the importance of C3 fragments in the shaping of the acquired immune repertoire [3].

The activation of C3 can also occur via the lectin pathway. In this pathway, targets of the lectin pathway can be recognized by the pattern-like recognition molecule mannan-binding lectin (MBL) or the Ficolins, which recognize specific carbohydrate moieties on lectin pathway activators. The binding of MBL or Ficolins to activators results in the activation of MBL-associated serum proteases like MASP-2, which then induce the activation of C4 and C2 and the generation of C4bC2a. This enzyme is the same as the one generated by the activation of the classical pathway. Next to the initiation of the CP by immune complexes also other agents like C-reactive protein (CRP), the long pentraxin-3 (PTX3), SIGN-R1 (a lectin that binds to microbial saccharides in the spleen and phosphatidyl serine that is exposed on apoptotic cells or on self-debris). While MBL reacts with mannose residues and sugars like N-acetyl-D-glucosamine, Ficolin-2 and Ficolin-3 recognize more specifically acetyl groups. Therefore these complement initiation/activation components can be regarded as specific soluble pattern recognition molecules that distinguish in the first place self from non-self and in addition can recognize ‘altered self’ such as modified self-tissue following apoptosis or modulation of the carbohydrate landscape on host tissue altered by reduced oxygen preside followed by re-oxygenation. This mechanism of complement activation could play an important role in activation of complement by apoptotic or necrotic tissue and amplify IgA-mediated renal damage.

A third pathway of complement is thought to be initiated by the slow and continuous turnover of the central component of complement C3. The initially generated C3b then reacts directly via its labile thio-ester with complement-activating structures. Once C3b is deposited on an activator, e.g. on specific microorganisms or cells and tissue, it can interact directly with factors B and D to form a labile C3bBb convertase (the amplification C3-convertase) that can cleave additional C3 molecules. This inherently labile C3bBb convertase can be stabilized by Properdin (P) which enhances the C3 activation potential of the C3bBb convertase and propels deposition of large numbers of covalently bound C3b molecules on the alternative pathway activator. Recent studies have indicated that next to direct binding of C3b to the activator, complement activation can be focused on a substance by Properdin itself. Elegant studies by Hourcade and colleagues have highlighted that Properdin can bind directly to a large number of bacteria [4]. Once Properdin is bound, and since P can easily polymerize, it can then recruit C3b and focus C3-amplification directly on pathogens or injured tissue and facilitate their interaction with cellular C3-receptors and subsequent elimination. It is therefore clear that the three pathways of the complement system are all initiated by a pattern-like recognition process involving soluble pattern-like molecules such as C1q, MBL, Ficolins and Properdin.

Independently of the initiation of complement activation, all three pathways and the two important convertases that are generated, i.e. C4b2a and C3bBb, lead to the most important steps in complement activation, namely activation of C3 into C3b and C3a. Once sufficient C3b is generated by any of the three pathways, the formation of the membrane attack complex C5b-C9, also called terminal complement complex (TCC) or membrane attack complex (MAC), can take place. Generation of the terminal complement pathway is associated with the formation of C5a which is a highly chemotactic fragment of C5a and which is additionally responsible for the activation of phagocytic cells like poly-morphonuclear leukocytes.

The C5b-9- complex was initially thought to be involved mainly in the cytoxic process of either foreign complement activating pathogens or host cells. More recent studies have shown that low numbers of C5b-9 induce activation of host cells leading to enhanced production of cytokines and chemokines and upregulation of adherence molecules such as LFA-1 [5]. Intermediate amounts of C5b-9 induce apoptosis of host cells and in this regard play a role in tissue turnover and homeostasis.

**Fig. 1:**
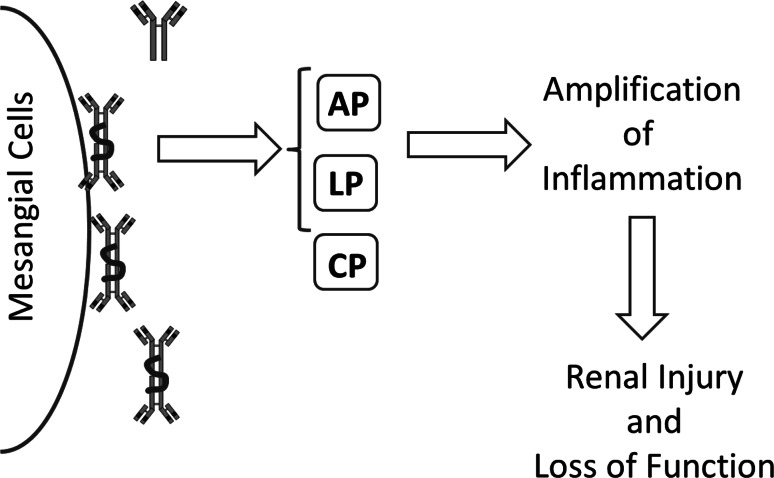
Complement in IgAN. Schematic view how deposition of high MW IgA deposited in the mesangial area of the kidney can activate the complement system through either the lectin or the alternative pathway. This will amplify the local inflammatory response and contribute to renal injury and loss of function. *AP* alternative pathway, *LP* lectin pathway, *CP* classical pathway, *MW* molecular weight, *IgA* immunoglobulin A

Activation of C3 results in the covalent binding of large numbers of C3b molecules to cells and tissue. This brings these cells into direct interaction with cellular C3b receptors (CR1, CD35). In plasma there are two regulators of C3 activation, namely factor I and factor H, that are able to convert C3b into iC3b. iC3b interacts strongly with C3-receptors like CR3. As mentioned, these receptors are found mainly on phagocytic cells such as macrophages and PMNs. Via these receptors iC3b bearing pathogens can be phagocytosed. During this process several inflammatory mediators are formed that can enhance the injury of cells and tissue (Fig. 1).

## Mechanisms of complement activation in IgAN

While deposition of IgA, mostly high molecular weight IgA1, in the renal mesangium is the hallmark of this disease, also deposition of C3 is a universal characteristic observed in these renal biopsies. Since IgG and C1q were observed only occasionally, the origin of this complement activation was originally linked mostly to the alternative pathway [6]. However, several recent findings have shed new light on this issue.

Following the notion that IgA, especially in its high molecular weight form, is able to activate complement via the lectin pathway, also mediators of the MBL pathway have been analysed [7, 8]. Indeed, MBL deposition has been observed in 20–25 % of cases, and cases with MBL deposition showed more renal injury and more proteinuria [9]. Several studies have now confirmed this finding, especially making use of C4d, a biomarker particularly well known in transplantation, where it is thought to be a more stable marker in tissue [8, 10]. In a large Spanish cohort, long-term prognosis in a 20-year follow up was significantly worse in those patients who showed C4d in their biopsy at the moment of diagnosis [11].

An intriguing unresolved issue is the general belief that altered O-linked glycosylation of IgA1 is a key step in the pathogenesis [12]. Although MBL is a lectin, it does not interact with the O-linked sugars. Therefore, the molecular explanation for interaction of MBL with IgA remains unclear. Based on this altered glycosylation, there is now compelling evidence that these altered IgA1 molecules can induce the formation of IgG autoantibodies directed against this deglycosylated hinge region. This could result in the formation of immune-complexes; however, it remains unclear how this contributes to complement activation in the kidney, since both C1q and IgG deposits are rare events. Interestingly we previously showed that renal deposits also contain secretory IgA in a small percentage of cases and that deposition of SIgA co-localizes with MBL and C4d [13]. This indicates that also other associated molecules (in this case the heavily glycosylated secretory component) can contribute to complement activation. The link with SIgA could be of importance, since SIgA is generated at mucosal surfaces and, clinically, flares of IgAN are associated with upper respiratory tract infections [14].

## A role for complement regulators

In recent years, initiated by the findings of large genetic genome-wide association study (GWAS) screening [15–17], attention has not only focused on the initiating routes of complement, but also on the regulators. Especially at the level of C3 there are multiple regulatory proteins, either membrane bound or in a soluble form. Important insights have been obtained in atypical hemolytic-uremic syndrome (aHUS) and other diseases related to uncontrolled alternative pathway activation, especially concerning the role of factor H and the factor H related proteins (CFHR) [18]. In GWAS analysis, it was found that deletion of CFHR1-CFHR3 was protective in IgAN [16]. These related molecules are considered a competitor for factor H and therefore might directly affect factor H function. However, precise understanding of how this deletion would impact IgAN is not clear [19]. Interestingly, the same deletion of this locus is seen as a risk factor for the development of aHUS and the development of anti-factor H antibodies [18].

## Concluding remarks

Over the past decade, there has been a growing interest in understanding the role of complement in the pathogenesis of IgAN. Several reasons have sparked this interest: identification of the lectin pathway as a possible contributor (with C4d as a biomarker), genetic analysis pointing towards alternative pathway regulation as a protective factor, and the availability of a therapeutic agent to interfere with the terminal route of complement activation. Irrespective of the route of activation, the effector mechanism of the terminal pathway is in common. Indeed C5b-9 deposition and appearance in the urine have been described [20]. In a recent case report, C5 blockade with eculuzimab was described [21]. Although an important observation, more research is required before complement inhibition can be more widely applied in the treatment of IgAN.
